# Longitudinal predictors for internalizing and externalizing symptomatology at age 4: KUNO-Kids cohort study

**DOI:** 10.3389/fpsyt.2024.1449108

**Published:** 2024-11-26

**Authors:** Irina Jarvers, Stephanie Kandsperger, Angelika Ecker, Susanne Brandstetter, Michael Kabesch, Angela Köninger, Michael Melter, Sebastian Kerzel, Jochen Kittel, Christian Apfelbacher, Romuald Brunner

**Affiliations:** ^1^ Department of Child and Adolescent Psychiatry and Psychotherapy, University of Regensburg, Regensburg, Germany; ^2^ University Children’s Hospital Regensburg (KUNO) at the Hospital St. Hedwig of the Order of St. John , University of Regensburg, Regensburg, Germany; ^3^ Research and Development, Wissenschafts- und Entwicklungs-Campus Regensburg (WECARE), Hospital St. Hedwig of the Order of St. John, Regensburg, Germany; ^4^ Department of Gynecology and Obstetrics, Hospital St. Hedwig of the Order of St. John, University of Regensburg, Regensburg, Germany; ^5^ Institute of Social Medicine and Health Systems Research, Otto von Guericke University Magdeburg, Magdeburg, Germany

**Keywords:** internalizing, externalizing, risk factors, protective factors, mental health, preschool children, longitudinal

## Abstract

**Introduction:**

Numerous early-life risk factors are thought to significantly contribute to the development of psychological problems in toddlerhood. However, these factors have seldom been investigated concomitantly and longitudinally, and few studies include both mothers and fathers. This study examines the longitudinal impact of early environmental, parental, and child-specific risk factors on children’s internalizing and externalizing symptomatology at age 4.

**Methods:**

Families were recruited from a perinatal center at birth and completed self-report questionnaires at birth, 4 weeks postpartum, 6 months postpartum, and annually thereafter. The final population-based sample consisted of *n* = 560 mothers (and fathers) who gave birth after June 2015, with children who turned 4 years old before March 31, 2021. The primary outcomes, children’s internalizing and externalizing symptomatology at age 4, were measured using the Strengthsand Difficulties Questionnaire. Linear mixed effect models were used to estimate growth curves for predictors between 4 weeks and 4 years postpartum, which were subsequently entered into multivariable linear regressions to predict internalizing and externalizing symptomatology at age 4.

**Results:**

The study identified several key risk factors: environmental (lack of social support, lower parental education, male sex), parental (poor parental mental health, increased parenting stress, parental sleep difficulties) and child-specific (children’s low physical health, children’s reduced sleep quality, temperament).

**Discussion:**

The findings underscore that most identified risk factors are related to children’s temperament, mental and physical health of parents, their experienced stress, and families’ social support networks. These insights highlight the importance of targeted interventions focusing on improving parental mental health, reducing stress, and enhancing social support to mitigate early-life psychological problems in children.

## 1 Introduction

Mental health disorders are among the leading causes of morbidity worldwide, with a global prevalence of 10.7% for any disorder (1). These issues often begin in childhood and adolescence (2), and can predict mental health disorders in adulthood (3–5). Mental health problems prior to diagnosed disorders, including internalizing (directed towards the self, e.g., anxiety, depression) and externalizing (directed towards the outside, e.g., aggression, hyperactivity) symptomatology, in early childhood can significantly contribute to mental health problems 14 and 16 years later (6, 7). This emphasizes the importance of early screenings, interventions, and the identification of risk factors contributing to internalizing and externalizing symptomatology in preschool children.

The dual-factor model of mental health posits that mental health is characterized by two separate but related dimensions: the presence of positive mental health and the absence of mental illness (8). This model emphasizes that individuals can experience a spectrum of well-being, even while suffering from mental health disorders. Consequently, when examining factors influencing mental health, it is essential to consider both those that increase the likelihood of developing mental health issues and those that offer protective benefits. In their systematic review, Carneiro et al. (9) identified three categories of risk factors for internalizing and externalizing symptoms in children aged 3 to 6 years: environmental, parental, and child-specific factors. Environmental factors include low socio-economic status (SES) and low parental education, both of which are linked to additional stressors like financial struggles (9). Parental factors encompass substance abuse and mental health issues, which can hinder a parent’s ability to attend to their child’s needs (10). Child-specific factors included male sex and temperament traits such as inhibitory control (11–14). Boys are often noted to have reduced deliberate control of attention compared to girls, making male sex a relevant risk factor (15). Additionally, temperament traits can influence behavior, leading to difficulties in inhibiting actions, reduced sociability, or increased shyness in children (16, 17). However, while these risk factors have been examined individually across various samples, the complex interplay among them remains poorly understood. It is crucial to also consider protective factors—such as supportive social networks—that can mitigate the negative impacts of these risk factors. By exploring both sides of the equation, it is possible to develop a more holistic understanding of child mental health.

In addition to meta-analyses, individual studies have highlighted additional risk factors, including parenting stress (18), parental physical health (19), and reduced quality of maternal bonding (20). Parenting stress and physical health problems can diminish the resources available for caring for children (18, 19), similar to the effects of mental health problems. Maternal bonding is considered a critical first step for fostering healthy attachment, as attachment problems are commonly linked to increased rates of psychopathology in children (21, 22). Lastly, social support is considered vital for parents, especially those with high SES and lower risk (23). It is believed to enhance the overall environment for parents, enabling them to seek help during periods of increased parental stress or mental health challenges (24, 25). However, most of these investigations have been conducted cross-sectionally and considered only a few known risk-factors. A comprehensive longitudinal study that examines these factors simultaneously would provide valuable insights.

In addition to postnatal risk factors, prenatal factors such as prenatal stress (26), toxicant exposure, and maternal infections (27) have been implicated. These prenatal factors are hypothesized to contribute to changes in brain development, which can subsequently impact children’s mental health and behavioral problems (27, 28). While the evidence linking these prenatal factors to child development is expanding, further research is essential to elucidate the specific mechanisms involved and how these influences interact with postnatal factors to shape mental health trajectories. It is particularly important to examine whether these prenatal influences continue to have predictive value as children age.

Sleep quality is another risk factor affecting both parents and children. Associations have been identified between sleep difficulties and mental health problems in children (29) and between sleep difficulties in mothers and parenting stress (30, 31). Additionally, extensive crying before the age of 1 due to abdominal pain, defined as colic, has shown mixed results regarding its impact on externalizing symptoms (32). Despite these associations, there is a notable research gap regarding the mechanisms through which sleep quality influences child development and whether it remains an influencing factor when considering associated risk factors, for example parental stress.

While numerous risk factors influencing children’s mental health have been examined in isolation, it is crucial to study them in conjunction to understand their potential interactions and collinearity. For instance, the interplay between prenatal and postnatal factors, including maternal sleep quality, stress, and environmental influences, can significantly shape developmental trajectories. Additionally, protective factors, including supportive parenting practices, strong social networks, and individual resilience traits, may mitigate the negative impacts of risk factors (33). Understanding how these protective elements interact with risk factors can provide a more comprehensive view of child mental health. The dynamic nature of these influences underscores the need for longitudinal studies, which are essential for exploring these complex relationships over time. Such studies can capture how risk and protective factors interact and change as children develop, revealing how they collectively affect mental health outcomes.

Moreover, the existing literature predominantly focuses on maternal influences, raising questions about the inclusion of paternal factors in research on children’s mental health. Recent meta-analyses suggest that father-specific aspects, such as attachment, also play a significant role in predicting children’s internalizing and externalizing symptoms (34, 35). This highlights an important gap in current research: the need to integrate paternal perspectives and experiences, alongside maternal influences, to fully understand the dynamics of child development and mental health. Addressing this gap will enhance our understanding of the multifaceted nature of these risk factors and inform more effective interventions.

To address this research gap, the present study utilized data from the KUNO-Kids health study (36), a German birth cohort, to investigate a range of environmental, parental (both maternal and paternal), and child-specific risk and protective factors for internalizing and externalizing problems at age 4. By adopting this comprehensive approach, the study aims to shed light on the intricate interactions among these factors, ultimately contributing to a deeper understanding of their collective impact on child mental health.

## 2 Methods

### 2.1 Study setting

**Figure 1:**
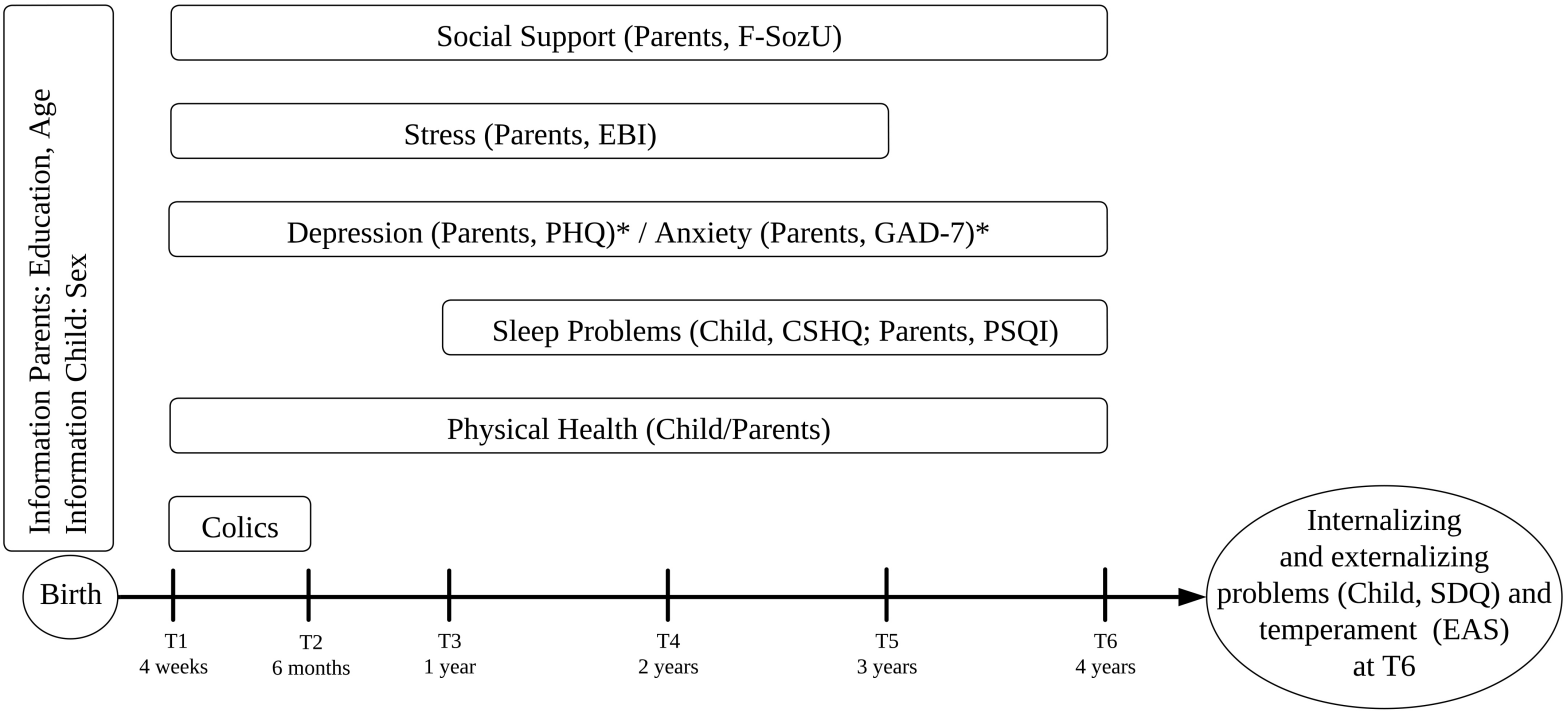
Overview of measures over time. * PHQ and GAD-7 were not filled out at T5. T2 was only filled out by mothers. PSQI, Pittsburgh Sleep Quality Index; FSozU, Social Support Questionnaire; GAD-7, Generalized Anxiety Disorder Scale; PHQ, Patient Health Questionnaire; EBI, Parenting Stress Index; CSHQ, Children’s Sleep Habits Questionnaire; SDQ, Strengths and Difficulties Questionnaire; Physical Health, general health assessment; Colics, assessment of colic at 4 and 6 months of age; EAS, Emotionality-Activity-Sociability Temperament Inventory.

The present study was conducted within the scope of the KUNO-Kids birth cohort study (36), which began recruitment in June 2015. The KUNO-Kids birth cohort study has three main objectives: (a) to enhance understanding of child health using novel omics technologies and a systems medicine approach, (b) to identify new modifiable factors affecting child health and opportunities for prevention, and (c) to provide a platform for evaluating the feasibility and effectiveness of targeted interventions. After childbirth, mothers were informed about the study’s objectives and procedures. Upon providing written, informed consent, they were included in the baseline assessment and subsequently contacted at 4 weeks (T1), 6 months (T2), and annually postpartum (T3-T6). The initial assessment after childbirth included an interview and a self-report questionnaire. All subsequent assessments were self-report postal questionnaires inquiring about the mother, the father, and the child (see **Figure 1** for an overview).

### 2.2 Ethical considerations

The authors assert that all procedures contributing to this work comply with the ethical standards of the relevant national and institutional committees on human experimentation and with the Helsinki Declaration of 1975, as revised in 2013. All procedures involving human subjects were approved by the Ethics Committee of the University of Regensburg (file numbers: 14-101-0347, 19-1646-101).

### 2.3 Participants

Participants were mothers who gave birth at St. Hedwig hospital in Eastern Bavaria (Regensburg, Germany) and their families. To be eligible for participation, mothers had to be at least 18 years old and possess adequate German comprehension to provide informed consent. Only one child per family was included in the study. A total of 1,528 families consented to participate between June 2015 and March 2021. At 4 years postpartum, measures for internalizing and externalizing symptomatology, as well as child temperament were introduced, resulting in a final sample of 560 families (36.6%). Attrition bias due to loss to follow-up is common in longitudinal studies and can lead to over- or underestimation of effects, especially with differential loss. In the KUNO-Kids study, a substantial percentage of families were lost between childbirth and the 1-year follow-up (36). Over time, the study increasingly overrepresents well-educated families, leading to greater selection bias. While this bias may not affect all exposure-outcome associations, it is particularly relevant where differential loss to follow-up occurs.

### 2.4 Measures

**Figure 1** provides an overview of the concepts and respective measures utilized in this study. The primary outcome measure was children’s externalizing and internalizing symptomatology at age 4.

#### 2.4.1 Environmental factors

Several environmental factors were assessed, including parental education, at-risk pregnancies (yes/no), antibiotics during pregnancy (yes/no) and premature birth (yes/no). Education was assessed via three categories, <10 years of education (scored as 1), 10-12 years of education (scored as 2) and a-levels and higher (scored as 3). Additional environmental risk factors, such as prenatal infections (*n* = 2), were excluded due to insufficient case numbers.

Mothers also completed the short form of the Fragebogen zur Sozialen Unterstützung (F-SozU) (37), a German measure of perceived social support. The F-SozU consists of 14 items, with scores ranging from 14 to 70, where higher values indicate a stronger social support network. Psychometric properties of the F-SozU were shown to be satisfactory in general population and clinical samples (38). In the current sample the F-SozU showed a reliability of Cronbach’s α = .96.

#### 2.4.2 Parental factors

Parental overall health was assessed via a single question asking parents to rate their health on a visual analogue scale (VAS) from 0 (worst possible health state) to 100 (best possible health state).

Parental sleep difficulties were assessed using the Pittsburgh Sleep Quality Index (PSQI) (39), a sleep report questionnaire measuring sleep quality over the past month. The PSQI score ranges from 0 to 21, with values below 6 indicating good sleep quality. Excellent psychometric properties have been established for both, samples in the Unites States (40), as well as Germany (41). The PSQI was administered starting at T3. In the current sample the PSQI showed a reliability of Cronbach’s α = .79.

Anxious and depressive symptomatology was measured using the Generalized Anxiety Disorder 7 (GAD-7) (42) and the Patient Health Questionnaire (PHQ) (43). The GAD-7 consists of 7 items with values between 0-4 corresponding to no or low, 5-9 to mild, 10-14 to moderate and 15-21 to severe anxiety symptoms. Psychometric properties including measurement invariance of the GAD-7 were confirmed to be excellent in a large German general population sample (44).The PHQ-9 consists of 9 items, with scores of 0-4 indicating none or minimal depression, 5-9 indicating mild depression, 10-14 indicating moderate depression, 15-19 indicating moderately severe depression, and 20-27 indicating severe depression. Reliability and validity of the German version of the PHQ-9 has been confirmed to be excellent in a large general population sample (45). Both questionnaires were administered at T1, T3, T4 and T6 for both parents and additionally at T2 for mothers. In the current sample the GAD-7 showed a reliability of Cronbach’s α = .86 and the PHQ-9 a reliability of Cronbach’s α = .91.

Parenting stress was assessed using the Eltern-Belastungs-Inventar (EBI) (46), the German version of the Parenting Stress Index (PSI) (47). Administered scales for both mothers and fathers included personal burden (PB), perceived lack of parental competence (PC), and attachment problems (AT). Internal consistency of the EBI was reported to be.95 and its construct validity could be confirmed (46). The questionnaire scores range from 4 to 20 points, with higher scores indicating greater parental stress. In the current sample the EBI showed a reliability of Cronbach’s α = .90.

#### 2.4.3 Child-specific factors

Children’s sleep difficulties were assessed using the German version of the Children’s Sleep Habits Questionnaire (CSHQ) (48), starting at T3. The CSHQ has 45 items, with 33 contributing to a total score. The clinical cut-off is set at 41, with scores above this threshold indicating clinically relevant sleep problems (49). Psychometric properties of the German version have been confirmed in community, as well as clinical samples with sleep disorders (48). In the current sample the CSHQ showed a reliability of Cronbach’s α = .85.

Internalizing and externalizing symptomatology were assessed using the Strengths and Difficulties Questionnaire 4-17 (SDQ) (50), which can compute both internalizing and externalizing scales. Scores can be categorized as slightly above average (80th-90th centile), high (90th-95th centile) and very high (<95th centile) (51). According to cut-offs introduced by Goodman et al. (51), scores of 7 are considered at-risk, and scores of 9 are critical. The psychometric properties of the German parent-report version of the SDQ have been reported as good (52). In our current sample, the SDQ demonstrated a total scale reliability of Cronbach’s α = .63, with reliabilities of Cronbach’s α = .67 for the internalizing scale and Cronbach’s α = .79 for the externalizing scale.

Child temperament was assessed using the German version of the Emotionality-Activity-Sociability Temperament Inventory (EAS) (53). The EAS includes 20 items across four scales: emotionality, activity, shyness, and sociability. Each item is scored from 1 to 5, with higher scores indicating greater agreement with the corresponding temperament dimension. The psychometric properties of the German EAS were previously validated in a sample of twins aged 2 to 14 years (54). In our current sample, the EAS demonstrated a total scale reliability of Cronbach’s α = .58, with individual scale reliabilities of α = .77 for emotionality, α = .70 for activity, α = .80 for shyness, and α = .61 for sociability. The SDQ and EAS were only administered at T6, with the SDQ serving as the outcome measure. Additionally, a variable differentiated between children who never showed colic and those who exhibited colic at 4 weeks and/or 6 months of age.

### 2.5 Statistical analysis

Data preparation and computation of growth curves were performed using the R statistical package, version 4.2.3 (55). Descriptive statistics, multiple imputation, and multivariable regression models were computed using SPSS 28 (56). The sample size was determined based on completed SDQ reports at T6 when children turned 4 years old. Missing data were present in 73.3% of variables, 20.9% of cases, and in 2.0% of values overall, primarily due to attrition. Little’s Missing Completely At Random test (MCAR) was conducted across all variables and time points and was non-significant (*χ* ^2^ = 1004.56, *p* = .620). Multiple imputation under fully conditional specification was used to create 20 imputed data sets, with Rubin’s rules applied for pooling and default settings used. For sensitivity analysis, a complete-case analysis was performed and compared with the imputed data. Missing data for mothers’ education, children’s sex, and colic were not imputed, leading to reduced sample sizes in later regressions (*n* = 549 for internalizing, *n* = 536 for externalizing).

Following recommendations by Chen et al. (57), a two-stage mixed effects model was used to assess longitudinal risk factors for internalizing and externalizing symptomatology at the age of 4. Given that our dependent variable was assessed only once, while most predictors were longitudinal, traditional linear mixed-effects modeling was not applicable. This two-staged mixed model effectively integrates the strengths of growth curve analysis and linear regression, allowing us to use growth curves as predictors and evaluate both the slope and intercept of individual variables over time, which is crucial for understanding their impact on symptomatology. Its capacity to handle longitudinal data, accommodate individual differences, manage missing data, and reduce collinearity ensures a thorough and efficient identification of significant risk and protective factors while maintaining model parsimony.

First, longitudinally time-varying variables were modeled as a function of time using random slopes and intercepts, similar to the growth curve model approach proposed by Welten et al. (58). Growth curves were computed for the following variables: mothers’ sleep difficulties (PSQI), fathers’ sleep difficulties (PSQI), mothers’ and fathers’ overall health, mothers’ social support (F-SozU), mothers’ and fathers’ depression and anxiety (PHQ-9, GAD-7), mothers’ and fathers’ parenting stress (EBI), children’s physical health, and children’s sleep difficulties (CSHQ). Individual values for the slope (development over time) and intercept (intensity) for each variable were extracted and used as predictor variables.

Second, the resulting subject-specific best linear unbiased predictor (BLUP) estimates were used as predictors in two linear regression models (internalizing score, externalizing score), in addition to predictors assessed once. The intercept represents the general value of scores over time, while the slope corresponds to changes in scores over time. This method effectively handles collinearity, accommodates missing data, and reduces the number of variables in the linear regression model. To ensure parsimony, regression models were recomputed to include only significant predictors from the second step (i.e., reduced regression models). For all models VIF values were below the critical threshold of 5 (59), with the largest VIF value being 3.9. The false discovery rate (FDR) was applied to correct for multiple comparisons in reduced regression models, with the significance level set at *p* <.05.

## 3 Results

### 3.1 Sample characteristics

**Figure 2:**
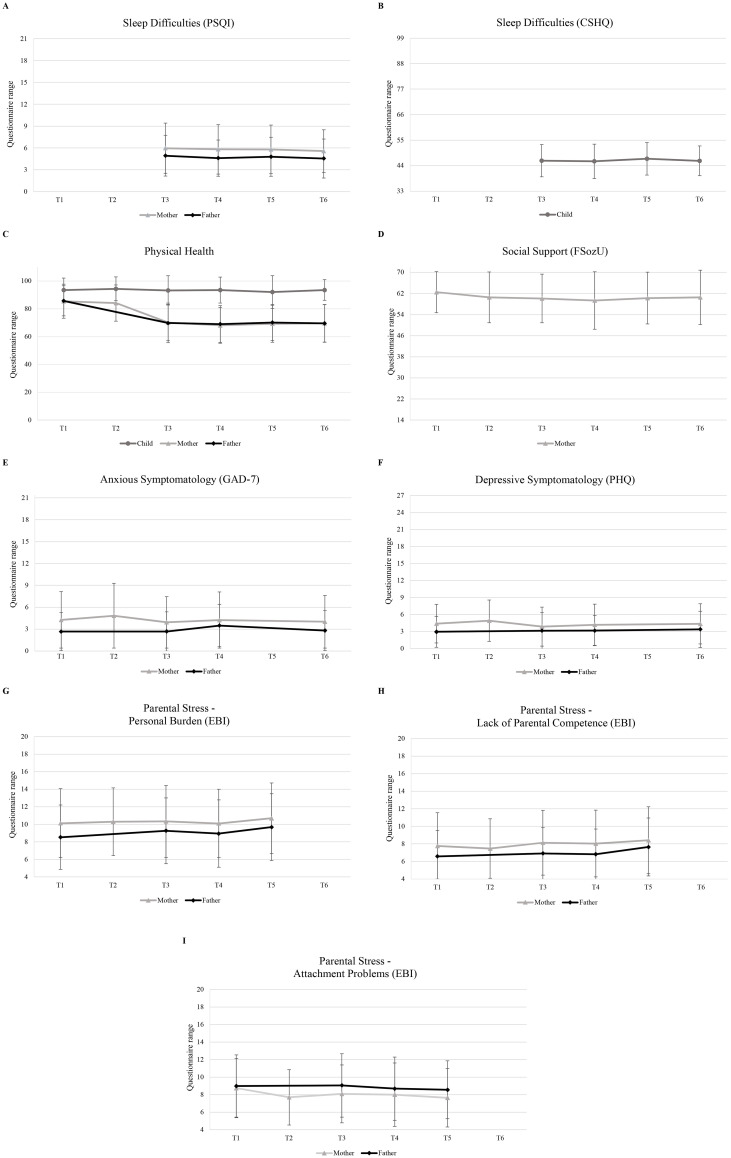
Mean questionnaire scores over time for mothers, fathers, and child. **(A)** Average sleep difficulties score (PSQI) across time for mothers and fathers, **(B)** Children’s average sleep difficulties score (CSHQ) across time. **(C)** Average physical health scores of mothers, fathers, and children across time. **(D)** Average social support reported by the mother across time. **(E)** Average anxious symptomatology (GAD-7) of mothers and fathers across time. **(F)** Average depressive symptomatology (PHQ) of mothers and fathers across time. **(G)** Average personal burden (EBI PB) reported by mothers and fathers across time. **(H)** Average lack of parental competence (EBI PC) reported by mothers and fathers. **(I)** Average attachment problems (EBI AT) reported by mothers and fathers. PSQI, Pittsburgh Sleep Quality Index; FSozU, Social Support Questionnaire; GAD-7, Generalized Anxiety Disorder Scale; PHQ, Patient Health Questionnaire; EBI, Parenting Stress Index; CSHQ, Children’s Sleep Habits Questionnaire; Physical Health, general health assessment.

The total sample consisted of 560 parents who completed the outcome measure SDQ for their 4-year-old children. Sample characteristics are depicted in **Table 1** and **Figure 2** depicts the non-imputed sample’s questionnaire scores over time.

**Table 1:** Demographic characteristics.

	Mothers	Fathers	Child
**Age in years**	32.4 (3.9)	35.6 (5.6)	
**Country of birth**	Germany 90.0 % Poland 1.4 % Other 8.6 %	Germany 89.5 % Kazakhstan 1.4 % Other 9.1 %	
**Education**			
< 10 years (%)	5.4 %	11.8 %	
10-12 years (%)	25.2 %	14.6 %	
a-levels (%)	67.7 %	50.5 %	
missing data (%)	1.8 %	23.0 %	
**GAD-7**	3.9 (3.3)	2.5 (2.7)	
**PHQ-9**	4.3 (3.5)	3.2 (2.9)	
**F-SozU**	60.55 (9.8)		
**PSQI**	5.8 (3.3)	4.7 (2.7)	
**EBI**			
Personal burden	10.3 (4.0)	9.1 (3.8)	
Parental competence	7.9 (3.7)	7.0 (3.1)	
Attachment	8.0 (3.3)	8.9 (3.5)	
**At-risk pregnancy**	39.5 %		
**Antibiotics during pregnancy**	21.4 %		
**Preterm birth**	5.6 %		
**SDQ**			
Internalizing			2.1 (2.2)
Externalizing			6.0 (3.6)
**EAS**			
Emotionality			2.9 (0.8)
Activity			4.1 (0.6)
Shyness			2.3 (0.8)
Sociability			3.5 (0.6)
**Colics**			
No colic (%)			76.4 %
Colic (%)			21.0 %
Missing (%)			2.5 %
**CSHQ**			46.3 (7.0)

PSQI, Pittsburgh Sleep Quality Index; FSozU, Social Support Questionnaire; GAD-7, Generalized Anxiety Disorder Scale; PHQ, Patient Health Questionnaire; EBI, Parenting Stress Index; CSHQ, Children’s Sleep Habits Questionnaire; SDQ, Strengths and Difficulties Questionnaire; Colics, assessment of colic at 4 and 6 months of age; EAS, Emotionality-Activity-Sociability Temperament Inventory.

For internalizing symptomatology on the SDQ at age 4, 12.1% of children were slightly above average (80th-90th percentile), 6.0% were high (90th-95th percentile), and 2.3% were very high (>95th percentile). For externalizing symptomatology, 13.8% of children were slightly above average, 5.3% were high, and 4.4% were very high. Regarding clinical cut-offs, a total of 5.1% of children were at-risk and 1.5% were critical for internalizing symptomatology, while 41.7% were at-risk and 23.8% were critical for externalizing symptomatology. Regarding comparisons with previously reported German norms for the SDQ, approximately 83.1% of children received a total score below 12, aligning with the reported percentage of 80.0 – 85.0% who show non-critical values (60). A total of 9.0% showed at-risk scores compared to norm percentages of 5.0 – 8.0% (60). Finally, 7.8% showed critically high scores, which aligns with the reported 7.0 – 10.0% (60).

### 3.2 Linear regressions predicting internalizing and externalizing symptomatology

Overall, two linear regression models were computed with the SDQ internalizing and externalizing scores as dependent variables (see **Tables 2**, **3**). Reduced regression models are depicted in **Table 4**. Longitudinal predictors included the intercept and slope of growth curves computed in the first step, along with children’s temperament. Mothers’ education and prenatal factors, such as at-risk pregnancies, premature birth and use of antibiotics during pregnancy, were added as predictors to both models. Sex and colic were added as predictors for the externalizing score, based on prior work (18, 32).

**Table 2:** Results of complete linear regression model predicting children’s internalizing symptomatology at age 4.

Dependent Variable	Predictor	*B*	SE	*B* CI		*t*	*P*	*R^2^*
				*Lower*	*Upper*			
SDQ Internalizing	**EAS Emotionality**	**0.58**	**0.10**	**0.37**	**0.78**	**5.50**	**<.001**	.47
	EAS Activity	0.10	0.13	-0.16	0.36	0.77	.444	
	**EAS Shyness**	**0.87**	**0.12**	**0.63**	**1.12**	**7.04**	**<.001**	
	**EAS Sociability**	**-0.60**	**0.14**	**-0.88**	**-0.31**	**-4.14**	**<.001**	
	PSQI Intercept (Mother)	-0.01	0.07	-0.14	0.12	-0.13	.897	
	PSQI Slope (Mother)	-2.49	1.79	-6.01	1.03	-1.39	.165	
	PSQI Intercept (Father)	-0.01	0.06	-0.12	0.10	-0.24	.810	
	PSQI Slope (Father)	-0.06	0.27	-0.59	0.47	-0.23	.821	
	Physical Health Intercept (Mother)	-0.19	0.21	-0.60	0.23	-0.88	.381	
	Physical Health Slope (Mother)	-0.15	0.36	-0.86	0.56	-0.41	.680	
	Physical Health Intercept (Father)	-0.20	0.15	-0.49	0.10	-1.30	.195	
	Physical Health Slope (Father)	0.67	0.43	-0.18	1.52	1.54	.124	
	FSozU Intercept	-0.02	0.02	-0.05	0.02	-0.98	.326	
	**FSozU Slope**	**-0.32**	**0.15**	**-0.61**	**-0.02**	**-2.08**	**.038**	
	**GAD-7 Intercept (Mother)**	**-0.16**	**0.07**	**-0.29**	**-0.03**	**-2.36**	**.019**	
	GAD-7 Slope (Mother)	0.51	0.73	-0.91	1.94	0.71	.479	
	**GAD-7 Intercept (Father)**	**0.22**	**0.10**	**0.01**	**0.42**	**2.10**	**.036**	
	GAD-7 Slope (Father)	-1.71	1.45	-4.55	1.13	-1.19	.236	
	**PHQ Intercept (Mother)**	**0.18**	**0.06**	**0.06**	**0.30**	**3.06**	**.002**	
	PHQ Slope (Mother)	0.07	0.39	-0.70	0.84	0.18	.857	
	PHQ Intercept (Father)	-0.05	0.07	-0.18	0.08	-0.76	.450	
	**PHQ Slope (Father)**	**1.13**	**0.37**	**0.40**	**1.86**	**3.05**	**.002**	
	**EBI PB Intercept (Mother)**	**-0.10**	**0.04**	**-0.18**	**-0.01**	**-2.27**	**.024**	
	EBI PB Slope (Mother)	-0.11	0.18	-0.46	0.25	-0.59	.553	
	**EBI PC Intercept (Mother)**	**0.11**	**0.05**	**0.00**	**0.21**	**1.97**	**.049**	
	EBI PC Slope (Mother)	0.40	0.34	-0.27	1.07	1.17	.242	
	EBI AT Intercept (Mother)	0.08	0.05	-0.02	0.18	1.63	.104	
	EBI AT Slope (Mother)	0.03	0.34	-0.63	0.69	0.09	.924	
	EBI PB Intercept (Father)	0.01	0.04	-0.07	0.09	0.26	.793	
	EBI PB Slope (Father)	0.09	0.26	-0.41	0.60	0.37	.711	
	EBI PC Intercept (Father)	0.08	0.07	-0.05	0.21	1.15	.249	
	EBI PC Slope (Father)	-0.01	0.29	-0.58	0.56	-0.03	.973	
	EBI AT Intercept (Father)	-0.05	0.05	-0.15	0.04	-1.08	.279	
	EBI AT Slope (Father)	0.26	0.62	-0.96	1.47	0.41	.681	
	Physical Health Intercept (Child)	0.00	0.02	-0.04	0.04	-0.06	.954	
	**Physical Health Slope (Child)**	**-0.30**	**0.13**	**-0.55**	**-0.05**	**-2.39**	**.017**	
	CSHQ Intercept (Child)	0.01	0.02	-0.03	0.04	0.35	.727	
	CSHQ Slope (Child)	-0.02	0.13	-0.28	0.25	-0.12	.901	
	Mother’s Education	-0.01	0.03	-0.07	0.05	-0.34	.733	
	Preterm birth	-0.05	0.34	-0.73	0.62	-0.16	.875	
	Antibiotics during pregnancy	0.17	0.19	-0.20	0.54	0.90	.370	
	At-risk pregnancy	0.08	0.16	-0.23	0.39	0.53	.596	

EAS, Emotionality-Activity-Sociability Temperament Inventory; PSQI, Pittsburgh Sleep Quality Index; FSozU, Social Support Questionnaire; GAD-7, Generalized Anxiety Disorder Scale; PHQ, Patient Health Questionnaire; EBI, Parenting Stress Index; PB, personal burden; PC, parental competence; AT, attachment; CSHQ, Children’s Sleep Habits Questionnaire; SDQ, Strengths and Difficulties Questionnaire.Significant predictors are marked in bold.

**Table 3:** Results of complete linear regression model predicting children’s externalizing symptomatology age 4.

Dependent Variable	Predictor	*B*	SE	*B* CI		*t*	*p*	*R^2^*
				*Lower*	*Upper*			
SDQ Externalizing	**EAS Emotionality**	**1.70**	**0.18**	**1.34**	**2.05**	**9.47**	**<.001**	.42
	**EAS Activity**	**1.14**	**0.23**	**0.69**	**1.59**	**4.96**	**<.001**	
	**EAS Shyness**	**-0.53**	**0.21**	**-0.95**	**-0.11**	**-2.49**	**.013**	
	EAS Sociability	-0.19	0.25	-0.69	0.30	-0.77	.439	
	**PSQI Intercept (Mother)**	**0.24**	**0.12**	**0.01**	**0.48**	**2.02**	**.043**	
	PSQI Slope (Mother)	4.99	3.09	-1.08	11.06	1.62	.107	
	PSQI Intercept (Father)	0.05	0.10	-0.14	0.24	0.55	.579	
	PSQI Slope (Father)	0.54	0.46	-0.36	1.44	1.17	.241	
	Physical Health Intercept (Mother)	-0.18	0.37	-0.89	0.54	-0.48	.632	
	Physical Health Slope (Mother)	-0.15	0.62	-1.37	1.07	-0.24	.807	
	Physical Health Intercept (Father)	-0.23	0.26	-0.73	0.28	-0.89	.375	
	Physical Health Slope (Father)	0.00	0.74	-1.45	1.46	0.01	.995	
	FSozU Intercept	-0.02	0.03	-0.07	0.04	-0.61	.541	
	FSozU Slope	-0.40	0.26	-0.91	0.11	-1.54	.123	
	GAD-7 Intercept (Mother)	0.03	0.12	-0.20	0.25	0.23	.817	
	GAD-7 Slope (Mother)	0.63	1.24	-1.81	3.07	0.51	.611	
	GAD-7 Intercept (Father)	0.12	0.17	-0.23	0.46	0.66	.507	
	GAD-7 Slope (Father)	-0.52	2.46	-5.36	4.31	-0.21	.832	
	PHQ Intercept (Mother)	-0.06	0.10	-0.26	0.14	-0.63	.532	
	PHQ Slope (Mother)	-0.92	0.67	-2.23	0.39	-1.38	.170	
	PHQ Intercept (Father)	-0.02	0.11	-0.24	0.21	-0.14	.885	
	**PHQ Slope (Father)**	**1.34**	**0.64**	**0.08**	**2.60**	**2.10**	**.037**	
	EBI PB Intercept (Mother)	-0.10	0.07	-0.24	0.04	-1.40	.163	
	EBI PB Slope (Mother)	-0.23	0.31	-0.83	0.37	-0.75	.456	
	EBI PC Intercept (Mother)	0.11	0.09	-0.08	0.29	1.14	.254	
	**EBI PC Slope (Mother**)	**1.16**	**0.58**	**0.02**	**2.31**	**2.00**	**.046**	
	**EBI AT Intercept (Mother)**	**0.18**	**0.09**	**0.00**	**0.35**	**2.01**	**.045**	
	EBI AT Slope (Mother)	-0.65	0.57	-1.77	0.47	-1.14	.256	
	EBI PB Intercept (Father)	-0.04	0.07	-0.17	0.10	-0.52	.605	
	EBI PB Slope (Father)	-0.05	0.44	-0.91	0.81	-0.10	.917	
	EBI PC Intercept (Father)	0.04	0.11	-0.19	0.26	0.32	.750	
	**EBI PC Slope (Father)**	**-1.19**	**0.50**	**-2.17**	**-0.21**	**-2.38**	**.018**	
	EBI AT Intercept (Father)	-0.06	0.09	-0.22	0.11	-0.65	.518	
	EBI AT Slope (Father)	1.66	1.06	-0.42	3.74	1.57	.117	
	Physical Health Intercept (Child)	-0.06	0.04	-0.14	0.01	-1.67	.095	
	Physical Health Slope (Child)	-0.17	0.22	-0.60	0.26	-0.78	.435	
	**CSHQ Intercept (Child)**	**0.07**	**0.03**	**0.01**	**0.14**	**2.33**	**.020**	
	CSHQ Slope (Child)	0.42	0.23	-0.03	0.88	1.84	.067	
	**Sex (Child)**	**0.81**	**0.27**	**0.29**	**1.34**	**3.03**	**.003**	
	Colics (Child)	0.62	0.33	-0.03	1.26	1.89	.060	
	**Mother’s Education**	**-0.16**	**0.05**	**-0.27**	**-0.06**	**-2.98**	**.003**	
	**Preterm birth**	**-1.21**	**0.59**	**-2.38**	**-0.04**	**-2.03**	**.042**	
	Antibiotics during pregnancy	0.21	0.32	-0.42	0.84	0.65	.514	
	At-risk pregnancy	-0.15	0.27	-0.68	0.39	-0.54	.589	

EAS, Emotionality-Activity-Sociability Temperament Inventory; PSQI, Pittsburgh Sleep Quality Index; FSozU, Social Support Questionnaire; GAD-7, Generalized Anxiety Disorder Scale; PHQ, Patient Health Questionnaire; EBI, Parenting Stress Index; PB, personal burden; PC, parental competence; AT, attachment; CSHQ, Children’s Sleep Habits Questionnaire; SDQ, Strengths and Difficulties Questionnaire.Significant predictors are marked in bold.

**Table 4:** Results of reduced linear regression models predicting children’s internalizing and externalizing problems at the age of 4.

Dependent Variable	Predictor	*B*	SE	*B* CI		*t*	*p*	FDR-*p*	*R^2^*
				*Lower*	*Upper*				
SDQ Internalizing	EAS Emotionality	0.68	0.09	0.50	0.86	7.31	<.001	.002	.45
	EAS Shyness	0.87	0.11	0.66	1.08	8.07	<.001	.002	
	EAS Sociability	-0.58	0.13	-0.84	-0.32	-4.33	<.001	.002	
	FSozU Slope	-0.41	0.12	-0.63	-0.18	-3.49	.001	.002	
	GAD-7 Intercept (Mother)	-0.17	0.05	-0.28	-0.07	-3.29	.001	.002	
	GAD-7 Intercept (Father)	0.13	0.05	0.02	0.23	2.33	.020	.024	
	PHQ Intercept (Mother)	0.23	0.05	0.13	0.33	4.55	<.001	.002	
	PHQ Slope (Father)	0.85	0.28	0.30	1.40	3.05	.002	.003	
	EBI PB Intercept (Mother)	-0.07	0.03	-0.14	0.00	-2.04	.042	.042	
	EBI PC Intercept (Mother)	0.13	0.04	0.05	0.22	3.01	.003	.004	
	Physical Health Slope (Child)	-0.27	0.12	-0.50	-0.03	-2.25	.025	.028	
SDQ Externalizing	EAS Emotionality	1.80	0.16	1.47	2.12	10.93	<.001	.002	.36
	EAS Activity	1.02	0.22	0.59	1.45	4.70	<.001	.002	
	EAS Shyness	-0.45	0.17	-0.79	-0.11	-2.59	.010	.017	
	PSQI Intercept (Mother)	0.13	0.06	0.02	0.25	2.30	.022	.031	
	PHQ Slope (Father)	1.71	0.49	0.75	2.68	3.50	.001	.002	
	EBI PC Slope (Mother)	0.58	0.43	-0.27	1.44	1.34	.180	.180	
	EBI AT Intercept (Mother)	0.19	0.06	0.07	0.31	3.10	.002	.004	
	EBI PC Slope (Father)	-0.87	0.38	-1.62	-0.12	-2.28	.023	.031	
	CSHQ Intercept (Child)	0.06	0.03	0.01	0.11	2.24	.026	.031	
	Sex (Child)	0.89	0.25	0.40	1.38	3.59	<.001	.002	
	Mother’s Education	-0.20	0.05	-0.29	-0.11	-4.25	<.001	.002	
	Preterm birth	-1.02	0.56	-2.11	0.08	-1.83	.068	.074	

EAS, Emotionality-Activity-Sociability Temperament Inventory; PSQI, Pittsburgh Sleep Quality Index; FSozU, Social Support Questionnaire; GAD-7, Generalized Anxiety Disorder Scale; PHQ, Patient Health Questionnaire; EBI, Parenting Stress Index; PB, personal burden; PC, parental competence; AT, attachment; CSHQ, Children’s Sleep Habits Questionnaire; SDQ, Strengths and Difficulties Questionnaire; FDR, false-discovery rate.

The model predicting the SDQ internalizing score was significant (*F* (42,525) = 10.08, *p* <.001) and explained 46.7% of the variance which corresponds to Cohen’s *f* = 0.94, indicating a large effect (61). Significant predictors included child temperament (EAS emotionality, shyness, sociability), social support over time (FSozU slope), mother’s and father’s anxious symptomatology (GAD-7 intercept), mother’s depressive symptomatology (PHQ intercept), mother’s perceived personal burden (EBI PB intercept), mother’s perceived lack of parental competence (EBI PB intercept), father’s depressive symptomatology over time (PHQ slope), and the child’s physical health over time (Health slope). In the reduced linear regression model, all predictors remained significant (see **Table 4**). The regression model on the complete-case dataset showed the same significant predictors.

The model predicting the SDQ externalizing score was significant (*F* (44,512) = 7.57, *p* <.001) and explained 41.6% of the variance, corresponding to Cohen’s *f* = 0.85, indicating a large effect. Significant predictors included child temperament (EAS emotionality, activity, shyness), mother’s sleep difficulties (PSQI intercept), mother’s perceived lack of parental competence over time (EBI PC slope), mother’s general attachment (EBI AT intercept), mother’s education, father’s depressive symptomatology over time (PHQ slope), father’s perceived lack of parental competence over time (EBI PC slope), the child’s overall sleep difficulties (CSHQ intercept), sex of the child and preterm birth. In the reduced linear regression model, mother’s perceived lack of parental competence over time (EBI PC slope) and preterm birth did not remain significant (see **Table 4**). The regression model on the complete-case dataset showed the same significant predictors.

## 4 Discussion

This study aimed to identify both general (sex, parental education, child temperament, prenatal factors) and longitudinal (sleep, social support, parental mental health, and stress) parental, environmental and child-specific risk and protective factors between the age of 4 weeks and 4 years for internalizing and externalizing symptomatology at the age of 4 within the KUNO-Kids cohort. Analyzing the prevalence of internalizing and externalizing symptomatology among 4-year-old children, we found that one-fifth exhibited above-average internalizing symptoms, while almost one-fourth displayed above-average externalizing symptoms according to the SDQ. This is in line with previously reported frequencies for internalizing and externalizing problems in German preschoolers (60).

Several risk and protective factors emerged that contribute to the early manifestation of these behaviors. For internalizing symptoms, maternal depression, fathers’ worsening depression and anxiety, mothers’ perceived lack of parental competence, and child emotionality and shyness were significant risk factors. In contrast, protective influences included increasing social support, maternal anxiety and perceived burden, improvements in children’s physical health, and child sociability. These results suggest that while certain aspects of parental mental health and child temperament may increase the likelihood of internalizing behaviors, social and emotional support networks, as well as improvements in child health, can mitigate these risks.

In terms of externalizing symptomatology, risk factors were largely related to maternal sleep difficulties, fathers’ increasing depression, perceived attachment problems, and child-related factors such as sleep difficulties, high emotionality, and activity, with male sex also posing a higher risk. Protective factors included higher maternal education, fathers’ perceived lack of parental competence, and child shyness. These findings suggest that externalizing behaviors are shaped not only by parental and child health but also by broader familial dynamics, indicating potential areas for intervention.

In the following, the identified risk and protective factors will be discussed in relation to findings from previous studies.

### 4.1 Environmental factors

The predictive impact of parental education on children’s externalizing symptomatology has been underscored in previous research, revealing that mothers with lower education levels are more susceptible to additional stressors such as unemployment, financial strain, or substandard housing conditions (9). In our study, we found that mothers’ education served as a significant negative predictor for externalizing symptomatology, though not for internalizing symptomatology. This discrepancy may be attributed to the generally high educational attainment within our sample and the nuanced presentation of internalizing symptoms in children (62). Additionally, externalizing symptomatology tends to be disruptive and may prompt the need for quick interventions, which can vary based on educational background.

Previous research has highlighted the positive influence of social support, particularly among mothers with high socioeconomic status and low-risk profiles (23). Given that our sample predominantly consisted of families with higher levels of education, the observed association between increased social support over time and reduced internalizing symptomatology in children at age 4 is plausible. However, it is important to acknowledge that while social support may mitigate internalizing symptoms within this demographic, its effectiveness may differ in a more diverse sample.

Overall, mothers’ education and perceived social support were protective factors for predicting children’s internalizing and externalizing symptomatology. The findings suggest that children of parents with lower educational status may benefit from targeted interventions that address the additional stressors these families face, such as unemployment and financial strain. Enhancing social support networks early on through parent-child centers, parent-child groups, and community programs could help parents connect with others in similar situations, fostering a supportive environment. These efforts may mitigate both externalizing and internalizing symptoms in children by providing parents with resources and peer support, reducing stress and promoting positive parenting practices.

### 4.2 Parental factors

Parental mental health, in particular maternal depression and anxiety, has frequently been identified as a crucial contributor to internalizing and externalizing symptomatology in children (10). Our study found that maternal depression and worsening paternal depression over time were significantly associated with increased internalizing symptoms in children. Interestingly, maternal anxiety was negatively related to children’s internalizing symptomatology, suggesting that anxious mothers reported fewer internalizing symptoms in their children compared to less anxious mothers. This could indicate a protective aspect of maternal anxiety, where heightened vigilance and attention to the child might play a role, or it might reflect a reporting bias.

Worsening paternal depression over time emerged as a consistent predictor for both internalizing and externalizing symptomatology in our sample. Previous research has similarly identified the impact of paternal depressive symptoms on both internalizing and externalizing problems in children (63, 64). Notably, it was the worsening of paternal depression, rather than elevated depression scores per se, that had a significant predictive effect. This worsening of depressive symptoms can disrupt the family dynamic, adding stress and burden to the other parent and potentially affecting the child’s emotional and behavioral development.

Another critical parental risk factor is parenting stress (9, 18), assessed through parents’ perceived personal burden, perceived lack of parental competence, and perceived attachment problems. In our study, mothers’ perceived attachment problems significantly predicted increased externalizing symptomatology in children, while mothers’ personal burden was a negative (protective) predictor of internalizing symptomatology. Personal burden reflects mothers’ attitudes towards their parental role and the degree to which they find it burdensome (65). An increased perception of the role as burdensome may lead to less attention toward the child and greater difficulty in recognizing internalizing symptoms (62).

Contrary to findings by Dagan et al. (35) and Deneault et al. (34), fathers’ attachment was not a significant predictor of either internalizing or externalizing symptomatology. Dagan et al. (35) argue that externalizing symptoms are more likely when attachment with both parents is disorganized. Given that mothers are more often the primary caregivers in Germany (66), it is possible that mothers’ perceived attachment problems had a stronger impact in our sample. Alternatively, fathers may have reported fewer attachment problems, or mothers’ reports of symptomatology may have been biased by their perceived attachment issues.

Regarding parental competence, mothers’ perceived lack of parental competence was a significant positive (risk) predictor of internalizing symptomatology, while fathers’ perceived lack of parental competence was a significant negative (protective) predictor of externalizing symptomatology. Mothers may feel incompetent in their role due to the often subtle and hard-to-detect nature of internalizing symptoms (62). Conversely, the relationship between fathers’ perceived parental competence and children’s externalizing behavior might be bidirectional. Children exhibiting externalizing behaviors may lead fathers to attribute the problem to the child’s behavior rather than their own competence. This relationship might also be influenced by the child’s temperament (13).

Finally, the impact of parental sleep difficulties was examined. Our study found that mothers’ sleep difficulties were significantly associated with increased externalizing symptomatology in their children. Sleep deprivation is known to lead to heightened exhaustion and overwhelm (30), which can impair mothers’ ability to effectively manage and respond to their children’s behavior. This reduced capacity to structure and guide behavior may contribute to more frequent and intense behavioral escalations in children.

Overall, parental mental health, parental stress, and maternal sleep difficulties played a crucial role in predicting children’s internalizing and externalizing symptomatology. The findings emphasize the critical need for parents, especially those experiencing maternal depression and worsening paternal depression, to access mental health care to support their emotional well-being and, in turn, their children’s development. Early screening for mental health difficulties among parents could help identify those who may need additional support, ensuring timely interventions. Promoting awareness of governmental aid programs can further empower parents by informing them of available resources, potentially reducing parental stress and alleviating fears of failure in their parenting roles. Additionally, addressing parenting stress and perceived parental competence through sleep support programs and community resources could foster a more nurturing environment for children, ultimately mitigating both internalizing and externalizing symptoms.

### 4.3 Child-specific factors

The most common child-specific factor associated with externalizing symptomatology is biological sex (9). Consistent with previous research, our study found that being male was significantly associated with increased externalizing symptomatology at age 4. One hypothesis for this association is that young boys may exhibit reduced deliberate control of attention (15), which can contribute to higher levels of disruptive and aggressive behaviors.

Additionally, children’s physical health from 4 weeks to 4 years of age was a significant negative (protective) predictor for internalizing symptomatology at the age of 4. While most studies highlight the predictive relationship between mental health problems in childhood and physical health issues in adolescence (67, 68), the bidirectional nature of this relationship has not been extensively examined. In adults, the impact of physical symptoms, such as bodily pain and gastrointestinal problems, on mental health is well-documented (69). It is plausible that physical symptoms similarly affect the mental well-being of young children.

Contrary to the findings of DeSantis et al. (32), our study did not confirm the presence of colic in the first year of life as a risk factor for externalizing symptomatology in 4-year-olds. While colic was a significant predictor in the main model, it did not remain significant in the more parsimonious, reduced model. This discrepancy suggests that colic might be a byproduct of other underlying difficulties and does not emerge as significant when multiple risk factors are considered concurrently. This finding highlights the importance of examining a broad range of factors to understand the complex interplay of influences on child development.

In addition to parental sleep difficulties, children’s overall sleep problems were significantly associated with externalizing symptomatology. Previous research has established links between childhood sleep problems and externalizing behaviors in adolescence (70). Our study reinforces these findings by highlighting the importance of sleep quality in early childhood, even when accounting for a wide range of concurrent factors. This underscores the critical need for early intervention and support to address sleep issues as a preventive measure against the development of externalizing symptomatology.

A significant child-specific factor explaining much of the variance in our study was child temperament. Child emotionality and shyness were positive (risk) predictors of internalizing symptomatology, while child sociability was a negative (protective) predictor. Additionally, child emotionality and activity were positive (risk) predictors, and child shyness was a negative (protective) predictor of externalizing symptomatology. Child emotionality consistently constituted a risk for both types of symptomatology in our sample, aligning with previous longitudinal studies from infancy/preschool to adolescence (16, 17). The dimension emotionality refers to children that frequently feel tension accompanied by intense autonomous arousal that evolves into feelings and expressions of fear and anger (71). This is also closely related to the concept of emotional reactivity, which is also known as a risk factor for emotion regulation difficulties (72), and subsequently mental health problems (73). Children with heightened emotionality may lack the skills to regulate these emotions, putting them at increased risk for mental health problems. Interestingly, child shyness had differential predictive power, with increased shyness relating to higher internalizing symptoms but lower externalizing symptoms. Shyness is commonly associated with internalizing traits (74) and a tendency to internalize thoughts and feelings (75), which may explain its relation to internalizing while inversely relating to externalizing behaviors. Additionally, child activity has been previously identified as a risk factor for externalizing problems (17). However, this relationship may be bidirectional, as hyperactive children might exhibit more movement, leading to higher reports of their activity level. Furthermore, child sociability was identified as a negative (protective) predictor of internalizing symptomatology, suggesting that children who are more sociable are less likely to exhibit internalizing symptoms. This finding aligns with numerous studies that highlight the positive impact of sociability on children’s mental health (76, 77). Considering that temperament is regarded as stable (78), it may be important to investigate which aspects of sociability, shyness and activity can be changed through interventions.

Overall, children’s temperament, sleep difficulties and physical health can be summarized as relevant child-specific factors for internalizing and externalizing symptomatology at the age of 4. The findings suggest the need for tailored interventions for boys, who are more prone to externalizing symptoms, emphasizing programs that enhance self-regulation, attention control, and behavioral management strategies. Prioritizing physical health through regular medical check-ups and prompt treatment of any health issues can play a crucial role in preventing emotional difficulties. Additionally, addressing sleep quality is essential, as sleep problems are linked to increased behavioral issues, highlighting the importance of educating parents on sleep hygiene. Fostering sociability and emotional regulation in children—especially those exhibiting high emotionality or shyness—can also help reduce the risk of internalizing symptoms. By implementing these targeted strategies, we can create a supportive environment that promotes positive mental health outcomes for children at risk, ultimately enhancing their overall development.

### 4.4 Strengths and limitations

The strengths of the present study include its longitudinal cohort design and relatively large sample size, which allowed for the comprehensive examination of risk and protective factors over an extended period. Additionally, the use of a two-model approach preserved subjective variance over time, reduced collinearity, and improved the handling of missing data. Additionally, our study stands out for its inclusion of data from both parents, enabling the identification of distinct paternal and maternal risk factors.

Limitations of the study include its reliance on self-reported assessments and the exclusive use of mothers’ reports to assess children’s internalizing and externalizing symptomatology at age 4. This may have led to an underrepresentation of the father’s perspective, potentially overlooking important aspects of the child’s behaviour. Future longitudinal studies would benefit from incorporating reports from multiple observers to obtain a more comprehensive understanding of children’s symptoms.

Additionally, the high attrition rate in the sample poses a limitation, resulting in a disproportionately educated sample and potentially limiting the generalizability of the findings to less educated populations. Finally, the absence of direct child variables, such as objective measures of development, is another limitation, as it precludes the control for confounding factors. Future research should include such measures to provide a more nuanced understanding of the factors influencing child behaviour.

## 5 Conclusion

Overall, our study identified several key risk and protective factors associated with children’s internalizing and externalizing symptomatology, encompassing environmental, parental, and child-specific domains. These factors include a lack of social support and lower parental education as environmental risks, reduced parental mental health and increased parenting stress as parental risks, and temperament, poor physical health, and sleep difficulties as child-specific risks. These findings underscore the critical importance of family support systems and early interventions targeting parental stress and mental well-being. Moreover, they highlight the complexity of addressing rather stable temperament traits such as children’s sociability and shyness. Moving forward, it is imperative for future research to investigate the effectiveness of parental interventions aimed at bolstering social support networks and addressing stress, and to assess their subsequent impact on children’s mental health outcomes.

## Data Availability

The raw data supporting the conclusions of this article will be made available by the authors, without undue reservation.

## The members of the KUNO-Kids study group

Andreas Ambrosch (Institute of Laboratory Medicine, Microbiology and Hygiene, Barmherzige Brüder Hospital, Regensburg, Germany), Petra A. Arndt (ZNL Transfercenter of Neuroscience and Learning, University of Ulm, Ulm, Germany), Andrea Baessler (Department of Internal Medicine II, Regensburg University Medical Center, Regensburg, Germany), Mark Berneburg (Department of Dermatology, University Medical Centre Regensburg, Regensburg, Germany), Stephan Böse-O’Reilly (University Children’s Hospital Regensburg (KUNO), Hospital St. Hedwig of the Order of St. John, Regensburg, Germany), Romuald Brunner (Clinic of Child and Adolescent Psychiatry, Psychosomatics and Psychotherapy, Bezirksklinikum Regensburg (medbo), Regensburg, Germany), Sara Fill Malfertheiner (Clinic of Obstetrics and Gynecology St. Hedwig, University of Regensburg, Regensburg, Germany), André Franke (Institute of Clinical Molecular Biology, Christian-Albrechts-University of Kiel, Kiel, Germany), Sebastian Häusler (Clinic of Obstetrics and Gynecology St. Hedwig, University of Regensburg, Regensburg, Germany), Iris Heid (Department of Genetic Epidemiology, University of Regensburg, Regensburg, Germany), Stefanie Heinze (Bavarian Health and Food Safety Authority (LGL) Munich, Germany), Wolfgang Högler (Department of Pediatrics and Adolescent Medicine, Johannes Kepler University Linz, Linz, Austria), Sebastian Kerzel (Department of Pediatric Pneumology and Allergy, University Children’s Hospital Regensburg (KUNO), Hospital St. Hedwig of the Order of St. John, Regensburg, Germany), Michael Koller (Center for Clinical Studies, University Hospital Regensburg, Regensburg, Germany), Michael Leitzmann (Department of Epidemiology and Preventive Medicine, University of Regensburg, Regensburg, Germany), Áine Lennon (Department of Conservative Dentistry and Periodontology, University Hospital Regensburg, University of Regensburg, Regensburg, Germany), David Rothfuß (City of Regensburg, Coordinating Center for Early Interventions, Regensburg, Germany), Wolfgang Rösch (Department of Pediatric Urology, University Medical Center, Regensburg, Germany), Bianca Schaub (Pediatric Allergology, Department of Pediatrics, Dr. von Hauner Children’s Hospital, University Hospital, LMU Munich, Munich, Germany), Stephan Weidinger (Department of Dermatology, Venereology and Allergy, University Hospital Schleswig-Holstein, Campus Kiel, Kiel, Germany) and Sven Wellmann (Department of Neonatology, University Children’s Hospital Regensburg (KUNO), Hospital St. Hedwig of the Order of St. John, Regensburg, Germany).
